# Case Report: PET/CT assessment of immunomodulatory therapy in anti-IgLON5 encephalitis with sleep apnea

**DOI:** 10.3389/fnins.2026.1720844

**Published:** 2026-02-23

**Authors:** Yan Liu, Huiling Luo, Xiaoxia Jia, Jing Lu, Ying Yang, Bing Fu, Jia Guo, Zheng Li

**Affiliations:** 1 Department of Neurology, Chengdu Fifth People’s Hospital, The Second Clinical Medical College, Affiliated Fifth People’s Hospital of Chengdu University of Traditional Chinese Medicine, Chengdu, China; 2 Department of Nuclear Medicine, Chengdu Fifth People’s Hospital, The Second Clinical Medical College, Affiliated Fifth People’s Hospital of Chengdu University of Traditional Chinese Medicine, Chengdu, China; 3 Department of Radiology, Chengdu Fifth People’s Hospital, The Second Clinical Medical College, Affiliated Fifth People’s Hospital of Chengdu University of Traditional Chinese Medicine, Chengdu, China

**Keywords:** 18F-FDG PET-CT, anti-IgLON5, apnea, autoimmune, case report, encephalitis, immunomodulatory therapy

## Abstract

Anti-IgLON5 disease is a rare autoimmune neurological disorder characterized by sleep disturbances, movement disorders, cognitive decline, bulbar symptoms, and respiratory dysfunction, with a complex interplay between autoimmunity and neurodegeneration. Recent studies have provided insights into its clinical manifestations, immunological mechanisms, and potential therapeutic approaches. However, there is limited research on the diagnosis of Anti-IgLON5 disease and the assessment of immunomodulatory treatment effects from a metabolic perspective by Fluorine-18 Fluorodeoxyglucose Positron Emission Tomography-Computed Tomography (18F-FDG PET-CT). This case describes a 59-years-old male presented with 1-year history of recurrent insomnia, low mood, staring spells, dysphagia, and choking while drinking water. Initially misdiagnosed with a gastrointestinal disorder or mental illness. The diagnosis was confirmed by positive serum and cerebrospinal fluid (CSF) tests for anti-IgLON5 antibodies, and 18F-FDG PET-CT showed abnormal FDG uptake in the medulla oblongata and left temporal lobe. After treatment with immunoglobulin pH4, mycophenolate mofetil tablets and corticosteroids significantly improved his condition. Follow-up at 3 months showed normalized metabolism on 18F-FDG PET-CT and improved sleep architecture. This case emphasizes the significance of anti-IgLON5 encephalitis in the differential diagnosis of complex sleep disorders, while also providing new insights and experiences regarding the evaluation of immune-modulatory therapy in the treatment of anti-IgLON5 encephalitis through 18F-FDG PET-CT. Overall, anti-IgLON5 disease is a multi-systemic condition that requires further research to identify precise and effective therapeutic strategies. Our case highlights the potential utility of 18F-FDG PET-CT in elucidating disease mechanisms and guiding clinical management.

## 1 Introduction

Anti-IgLON5 disease is an emerging neurological entity that bridges the realms of autoimmunity and neurodegeneration. First described in 2014, this disorder is characterized by a complex clinical presentation, including sleep disturbances, movement disorders, cognitive decline, and progressive neurodegeneration, with a strong association with autoantibodies targeting the neuronal cell adhesion molecule IgLON5 (Chen et al., 2020; Della Marca et al., 2014; Högl et al., 2015; Sabater et al., 2014). The discovery of these antibodies and their link to specific human leukocyte antigen (HLA) alleles has implicated an autoimmune etiology, although the exact pathogenic mechanisms remain elusive (Schiff et al., 2023). Recent studies have explored the clinical spectrum of anti-IgLON5 disease, revealing a range of manifestations from parasomnia and sleep-disordered breathing to severe cognitive and motor impairments (Brunetti et al., 2019). The identification of tau pathology in affected brain regions further complicates the understanding of this disorder, suggesting a dual role of autoimmunity and neurodegeneration in its pathogenesis (Lee et al., 2024; Ni et al., 2022; Theis et al., 2023). In parallel, research has focused on the potential therapeutic avenues, particularly immunotherapy, with varying degrees of success reported (Grüter et al., 2023).

More and more researches on anti-IgLON5 disease have characterized its clinical presentations, underlying mechanisms, and treatment responses. Movement disorders, sleep disturbances, bulbar dysfunction, and cognitive impairment are common initial symptoms. IgLON5 antibodies disrupt neuronal cytoskeletal integrity, linking autoimmunity to neurodegenerative processes. Genetic associations, particularly with the HLA-DRB1*10:01, HLA-DQB1*05:01 haplotype, support the autoimmune origin and neurodegenerative component of the disease (Gaig et al., 2019, 2021; Landa et al., 2020). Recent studies and case reports on anti-IgLON5 disease highlight its diverse clinical presentations, including movement disorders, sleep disturbances, cognitive impairment, and bulbar dysfunction. While immunotherapy has shown limited efficacy in many cases, early and aggressive treatment has been associated with significant clinical improvement in some patients. Additionally, spontaneous recovery and residual mild symptoms have been reported, indicating the potential for varied clinical courses. Neuroimaging and biomarkers may further elucidate disease progression and treatment response (Graus and Santamaría, 2017; Ramanan et al., 2018; Schöberl et al., 2018; Shambrook et al., 2021).

## 2 Case description

A 59-years-old male presenting with recurrent insomnia of unknown origin for 1 year, characterized by difficulty falling asleep, frequent nocturnal awakenings, and inability to return to sleep. Additional symptoms included persistent low mood, occasional staring spells accompanied by delayed reactions, intermittent dysphagia, and choking while drinking water. Initially, the patient consulted the Department of Gastroenterology and was misdiagnosed with a gastrointestinal disorder. Despite treatment, no significant improvement was observed. Subsequently, following psychiatric evaluation at our institution, the patient underwent standardized psychometric assessments. The Hamilton Anxiety Rating Scale (HAM-A) score of 21 (clinical significance threshold: ≥14) confirmed moderate-to-severe anxiety symptomatology, while the Hamilton Depression Rating Scale (HAM-D) score of 15 (interpretive range: 14–18 for moderate depression) suggested mild-to-moderate depressive features. A provisional diagnosis of mixed anxiety-depressive disorder was established. The patient was treated with olanzapine, alprazolam, tianeptine citrate, and venlafaxine. However, the response was suboptimal, and the symptoms progressively worsened. One week prior to admission, the patient’s wife observed new-onset delirious speech and hallucinations. The patient remained intermittently responsive and was able to provide accurate answers upon regaining lucidity, which prompted a visit to the department of neurology for further treatment.

**FIGURE 1:**
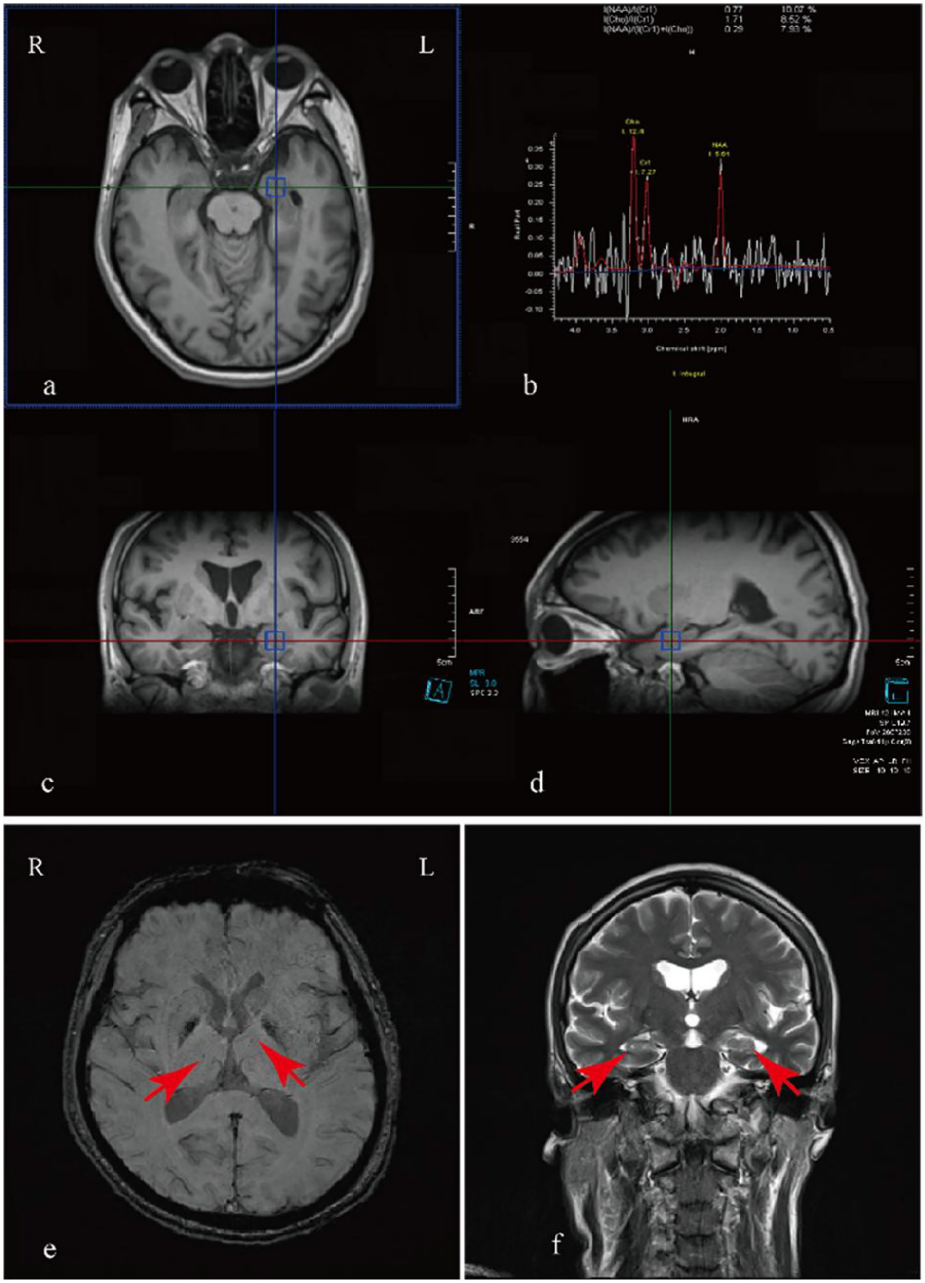
Magnetic resonance imaging findings. Cranial MRS **(a–d)** and MRI **(f)** demonstrated a reduction in the volume of the bilateral hippocampi, with more pronounced atrophy on the left side, along with widened bilateral choroidal fissures and mild dilation of the bilateral lateral ventricle horns. Cranial SWI **(e)** revealed minor, spotty, and patchy deposits of paramagnetic material in the bilateral basal ganglia regions.

Following admission, a comprehensive cranial Magnetic Resonance Imaging (MRI) indicated mild cerebral atrophy, reduced volume of the bilateral hippocampi with the left side being more pronounced, widened bilateral choroidal fissures, and mild dilation of the bilateral lateral ventricle horns. The signal in the bilateral hippocampi lacked uniformity. Cranial Magnetic Resonance Spectroscopy (MRS) showed a decreased N-acetylaspartate (NAA) to [choline (Cho) plus creatine (Cr)] ratio, with the right side at 0.28 and the left side at 0.29 (normal reference range: ≥0.72), which represents a reduction in bilateral hippocampal volume. Cranial Susceptibility Weighted Imaging (SWI) revealed minor spotty and patchy paramagnetic material deposits in the bilateral basal ganglia regions, and the bilateral colliculi appeared less distinct (Figure 1). Neuropsychological assessments revealed multi-domain cognitive impairment, evidenced by a Montreal Cognitive Assessment (MoCA) score of 13/30 (indicative of dementia-range dysfunction) and a Mini-Mental State Examination (MMSE) score of 20/30 (consistent with mild cognitive impairment). Significant functional decline was demonstrated through the Activities of Daily Living (ADL) scale, with the patient scoring 23/56, reflecting substantial dependency in routine tasks. The standardized Water Swallow Test (WST) demonstrated clinically significant dysphagia characteristics: The patient required three swallowing attempts to complete 90 mL water consumption without overt aspiration, meeting the diagnostic criteria for Grade 2 dysphagia according to the Fujishima classification system.

### 2.1 Polysomnography (PSG)

Polysomnography revealed an extended sleep latency period, post-sleep onset wakefulness totaled 263.0 min with 16 arousals, indicating poor sleep continuity and low sleep efficiency, total sleep time amounted to 15.0 min. The sleep architecture showed an increased proportion of stage 1 sleep, with absent stages 2, 3, and Rapid Eye Movement (REM) sleep.

**FIGURE 2:**
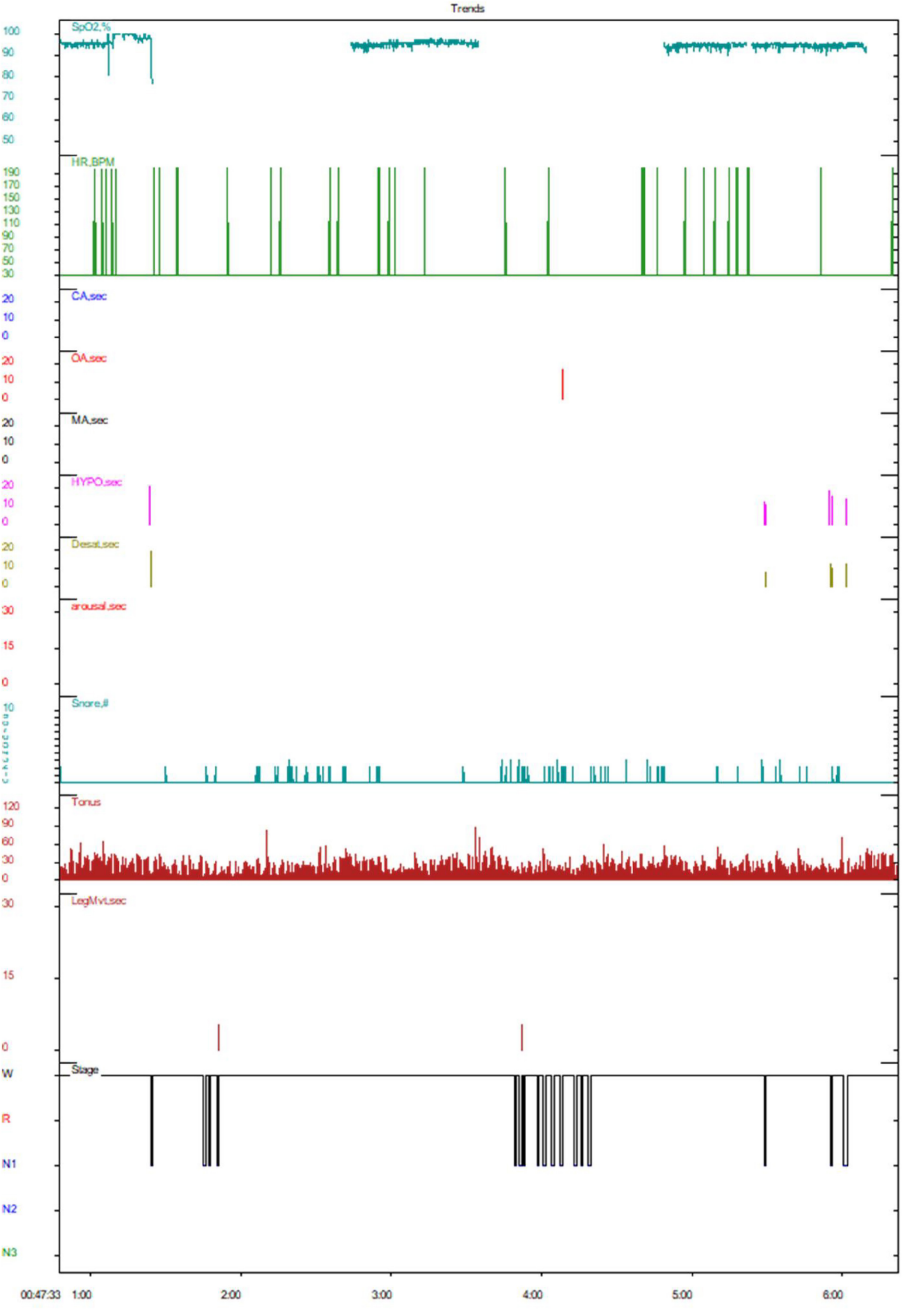
Polysomnography. CA, central apnea; OA, obstructive apnea; MA, mixed apnea; HYPO, hypopnea; arousal, awakening event; SpO2%, oxygen saturation; LegMvt, motor event; Pos, posture. As shown in the figure, this patient demonstrated poor sleep continuity and reduced sleep efficiency. The sleep architecture revealed an increased proportion of stage 1 sleep, with the absence of stages 2, 3, and REM sleep. Obstructive apneas were observed, accompanied by severe hypopnea during sleep.

Throughout the night, there were a total of 7 apnea episodes, including 1 obstructive apnea episode (longest lasting 18.0 s) and 6 hypopnea episodes (longest lasting 21.5 s), with no central or mixed apneas observed. The Apnea-Hypopnea Index (AHI) reached 24.0 events per hour (normal is less than 5 per hour), with associated oxygen desaturation index and time at 97.3 and 19.7 min per hour, respectively, and minimum and average oxygen saturation levels at 92% and 95%. Snoring time accounted for 11.3% of total sleep time. Collectively, these findings are consistent with the diagnostic criteria for “moderate sleep apnea hypopnea syndrome” (Figure 2). A 24-h long-term video electroencephalogram (EEG) revealed no abnormal brain waves, nor were there any typical interictal epileptiform discharges observed (Supplementary material).

### 2.2 Analysis of anti-IgLON5 antibodies

**FIGURE 3:**
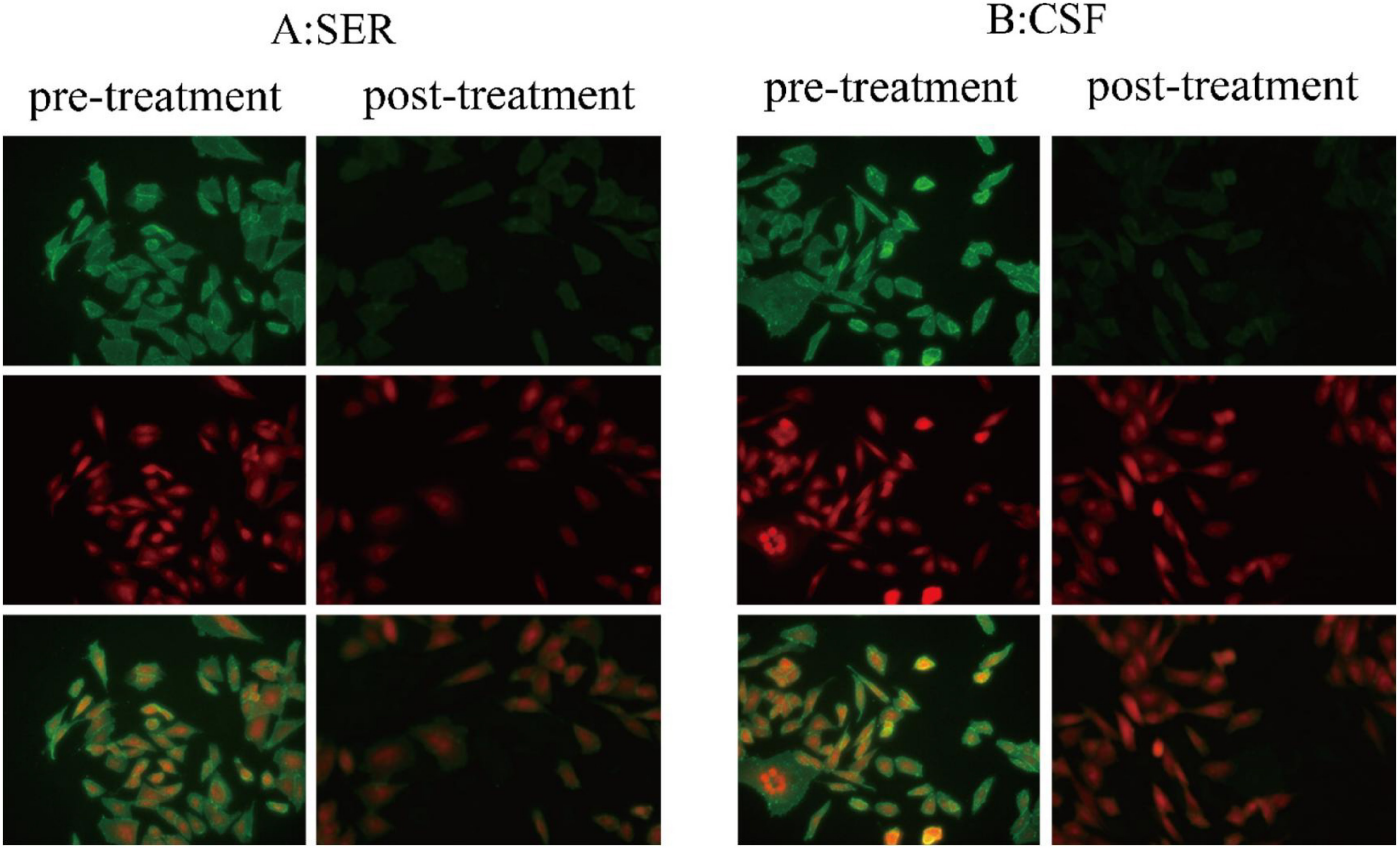
Autoimmune encephalitis antibody detection. **(A,B)** show strong positivity for anti-IgLON5 antibodies in the serum and CSF on cell-based assays. Additionally, after treatment, the titer of anti-IgLON5 antibodies significantly decreased. Green fluorescence: anti-IgLON5 antibodies. Cell-based assays (CBA) entail the transfection of target antigen genes into mammalian cells, facilitating the specific expression of corresponding antigens within these cells. During the transfection process, red fluorescent protein is co-expressed as an internal control to ensure accurate detection. Following transfection, the cells are immobilized onto the wells of a microplate to generate antigen slides. The principle of indirect immunofluorescence is subsequently employed for the semi-quantitative detection of specific antibodies in patient serum and CSF samples. The presence of distinct green fluorescence on the cell membrane of successfully transfected cells within the sample wells indicates antibody positivity.

Lumbar puncture revealed a CSF pressure of 100 mmH2O, with a protein level of 573.7 mg/L, and white blood cell (WBC) count of 3 × 10∧6/L. Immunological profiling demonstrated dual seropositivity for anti-IgLON5 antibodies with distinct compartmentalization: CSF analysis revealed an IgG antibody titer of 1:100 (threshold > 1:3.2), while serum quantification showed markedly elevated IgG levels at 1:3200(threshold > 1:10). This striking 32-fold disparity in antibody concentrations between serum and CSF compartments (serum/CSF ratio = 32:1) suggests either impaired blood-brain barrier integrity or potential intrathecal synthesis of specific antibody subpopulations (Figure 3).

**FIGURE 4:**
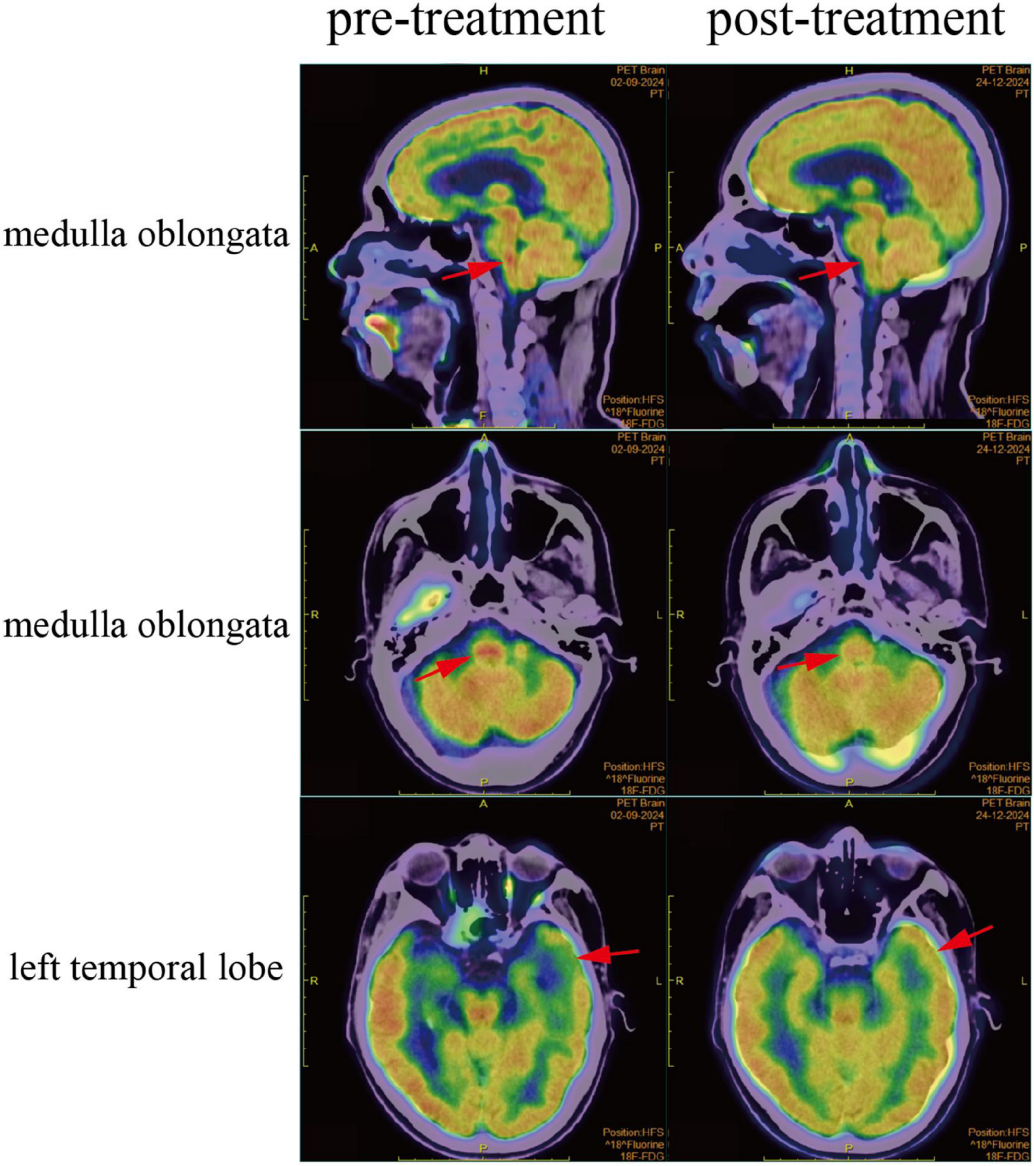
Fluorine-18 Fluorodeoxyglucose Positron Emission Tomography-Computed Tomography (18F-FDG PET-CT) scan. As illustrated, the 18F-FDG PET-CT scan reveals reduced FDG uptake in the left temporal lobe and increased FDG uptake in the medulla oblongata prior to treatment. Following treatment, both the medulla oblongata and the left temporal lobe regions have returned to normal metabolic activity.

Because of the patient’s recurrent symptoms of dysphagia and coughing while drinking water, further18F-FDG PET-CT was performed to elucidate the underlying pathology, and the results indicated a reduction in FDG uptake in the left temporal lobe and increased FDG uptake in the medulla oblongata (Figure 4). In addition, both serum p-Tau 181 and p-Tau 217 levels elevated. Sanger sequencing detected the presence of HLA-DRB1*10:01 and HLA-DQB1*05:01 alleles in peripheral blood human leukocyte HLA genes. In conclusion, the patient was ultimately diagnosed with anti-IgLON5 autoimmune encephalitis.

### 2.3 Treatment and outcome

The patient exhibited severe mental and behavioral abnormalities, making it impossible to proceed with plasma exchange. Therefore, after discussion with the patient’s families, we opted for human immunoglobulin for immunoregulation therapy. The initial dose was 0.4 g per kilogram of body weight per day, administered for five consecutive days. Subsequently, the regimen was adjusted to oral mycophenolate mofetil 0.5 g twice daily, oral prednisone acetate 60 mg daily (with a gradual weekly reduction of 5 mg for maintenance therapy), and olanzapine 5 mg orally every night to control mental symptoms. During oral corticosteroid therapy, the patient developed mild GI upset, including nausea, which resolved with symptomatic management. Furthermore, due to severe hallucinations and psychosis, the patient demonstrated poor treatment adherence. With coordinated support from both families and physician, the initial phase was successfully completed. After 10 days of treatment, the patient’s mental and behavioral abnormalities significantly improved. After treatment, the patient improved and was discharged from the hospital. Three months later, the patient was readmitted for follow-up examinations. The follow-up 18F-FDG PET-CT scan revealed that the previously observed areas of increased FDG uptake in the medulla oblongata and decreased FDG uptake in the left temporal lobe had both returned to normal metabolism (Figure 4). Meanwhile, the patient’s cognitive function significantly improved, and symptoms indicative of medulla oblongata dysfunction, such as dysphagia and coughing while drinking water, resolved.

A lumbar puncture was performed to assess the levels of anti-IgLON5 antibodies in the CSF and serum. The results indicated a significant reduction in antibody titers compared to previous measurements. Specifically, the anti-IgLON5 antibody titer in the CSF decreased from 1:100 to 1:32, while the serum titer decreased from 1:3200 to 1:320 (Figure 3). Additionally, a follow-up PSG showed that the patient’s sleep latency had shortened compared to before, with an increase in total sleep duration, and the proportion of stage 1, 2, 3 and REM sleep is roughly normal. During sleep, there were 11 episodes of REM sleep observed, and the apnea-hypopnea index (AHI) had decreased from 24 to 18.4 events per hour, average oxygen saturation increased from 95% to 96%, indicating significant improvement in the patient’s sleep and breathing compared to pre-treatment levels. During the 1-year post-discharge period, with regular outpatient follow-up every 1–2 months, the patient has maintained immunomodulatory therapy with mycophenolate mofetil. The patient reported substantial symptomatic improvement, characterized by the resolution of hallucinations, enhanced cognitive function, and sleep is now managed with only 2 mg of nightly eszopiclone without the need for olanzapine. Furthermore, he has regained the ability to undertake short outings independently. Overall, the patient’s clinical condition across various aspects demonstrated marked improvement. Table 1 illustrates all the diagnostic and therapeutic steps.

**TABLE 1:** Clinical signs, diagnostic tools, and treatment strategies timeline.

Time	Symptoms and treatment
Six months prior to admission	Difficulty falling asleep, low mood, slow response, occasional difficulty swallowing and choking when drinking water. The psychological department diagnosed it as “anxiety and depression state.” Treatment with olanzapine, alprazolam, tandospirone citrate and venlafaxine was ineffective
One week before admission	Raving nonsense, hallucinations, abdominal pain
D1	Insomnia, depression, absent-mindedness, slow response, difficulty in swallowing, choking when drinking water, and hallucinations: Admission
D2	Lumbar puncture, the titer of CSF anti-IgLON5 antibodies is 1:100, the titer of serum anti-IgLON5 antibodies is 1:3200, both increased
D3	Serum p-Tau 181/217 elevated, Sanger sequencing detected the presence of HLA-DRB1*10:01 and HLA-DQB1*05:01 alleles in peripheral blood human leukocyte HLA genes
D4	MRI: Mild cerebral atrophy with bilateral hippocampal volume reduction; MRS: Right NAA/(Cho + Cr) = 0.28, left NAA/(Cho + Cr) = 0.29; SWI: Few paramagnetic deposits in bilateral basal ganglia; 18F-FDG PET-CT: FDG hypometabolism in the left temporal lobe, FDG hypermetabolism in the medulla oblongata; IVIg therapy (Day 1); Olanzapine 5 mg nightly
D5	PSG: DIMS and SAHS; IVIg therapy (Day 2)
D6	VEEG: Normal; IVIg therapy (Day 3)
D7-8	IVIg therapy (Day 4–5)
D9-13	Mycophenolate mofetil 0.5 g twice daily, prednisone acetate 60 mg daily (with a gradual weekly reduction of 5 mg)
D14	Insomnia, low mood, blank stare, delayed response, difficulty swallowing, choking cough when drinking water, hallucinations, and other symptoms have improved; Discharged
Three months after discharge	Clinical symptoms improved; 18F-FDG PET-CT: normal; lumbar puncture, titer of anti-IgLON5 antibody in CSF decreased from 1:100 to 1:32, while the serum titer decreased from 1:3200 to 1:320; PSG: Sleep and breathing have significantly improved
One year after discharge	Mycophenolate mofetil orally; The symptoms have significantly improved

CSF, cerebrospinal fluid; HLA, human leukocyte antigen; MRI, Magnetic Resonance Imaging; MRS, Magnetic Resonance Spectroscopy; NAA, N-acetylaspartate; Cho, choline; Cr, creatine; SWI, Susceptibility Weighted Imaging; IVIg, Initiated intravenous immunoglobulin; 18F-FDG PET-CT, Fluorine-18 Fluorodeoxyglucose Positron Emission Tomography-Computed Tomography; PSG, Polysomnography; DIMS, disorder of initiating and maintaining sleep; SAHS, sleep apnea-hypopnea syndrome; VEEG, Video electroencephalogram.

## 3 Discussion

Anti-IgLON5 antibody-associated disease is an insidious and progressive central nervous system disorder that predominantly affects middle-aged and elderly patients, typically presenting with a subacute clinical course and exhibiting significant inter-individual variability in manifestations (Tagliapietra et al., 2021). Sleep disturbances are characteristic features of IgLON5 disease, encompassing rapid eye movement (REM) sleep behavior disorder, symptoms of medullary syndrome, non-REM sleep parasomnias, and sleep-disordered breathing (Honorat et al., 2017). Currently, there are no definitive diagnostic criteria or specific therapeutic regimens for this condition. The disease can impair central nervous system function, leading to irreversible neurological damage and even death (Grüter et al., 2023). Therefore, early recognition and diagnosis of anti-IgLON5 antibody-associated disease are of great importance. Moreover, immunomodulatory therapy has been shown to be effective in improving clinical symptoms and reducing mortality rates. Additionally, we have found that 18F-FDG PET-CT is of significant assistance in diagnosing and evaluating therapeutic outcomes, which has not been previously reported in the literature.

In the diagnosis of anti-IgLON5 antibody-associated disease, the detection of anti-IgLON5 antibodies in both serum and CSF is crucial for confirming the diagnosis. This case report provides a detailed description of a patient presenting with severe sleep disturbances and sleep apnea, with a disease course spanning over a year, repeatedly misdiagnosed, and progressively evolving into cognitive impairment, accompanied by dysphagia and psychiatric symptoms, severely affecting the patient’s daily life. The anti-IgLON5 antibodies were detected at high titers in both the CSF and blood of this patient. Research identifies HLA-DQ5 subtypes, particularly HLA-DQB1*05:01, as key mediators of risk in anti-IgLON5 disease, highlighting a stronger association with HLA-DQ than HLA-DR and suggesting a T cell-mediated autoimmune response to post-translationally modified IgLON5 peptides (Schiff et al., 2023). In this patient, the HLA-DRB11001 and HLA-DQB1*0501 alleles of the HLA gene in peripheral blood leukocytes were both positive. Dysfunction of IgLON5 caused by anti-IgLON5 antibodies may disrupt the protein’s interaction with the intracellular cytoskeletal network, leading to instability of the neuronal microtubule system and inducing hyperphosphorylation and accumulation of microtubule-associated protein tau, thereby causing neuronal dysfunction (Gelpi et al., 2016). The pathological aggregation of hyperphosphorylated tau protein, particularly in the hypothalamus and tegmentum of the midbrain, including key nuclei that regulate sleep, is thought to be associated with the significant sleep disturbances in patients with anti-IgLON5 disease. The patient we reported showed abnormal metabolism in both the medulla oblongata and the temporal lobes, abnormal signals in the hippocampal region, and elevated levels of p-Tau 181 and p-Tau 217 in the blood.

Current data regarding immunotherapy are limited, and its efficacy remains a matter of debate. However, recent studies have described a favorable response to this treatment (Lee et al., 2024). Autoimmune mechanisms are thought to play a primary role at least in the initial stages of anti-IgLON5 disease, and early immunotherapy administered prior to the onset of neurodegeneration is associated with better long-term clinical outcomes (Grüter et al., 2023). In this case, following a 5-days course of intravenous immunoglobulin therapy, there was an improvement in the patient’s psychiatric symptoms and sleep disturbances. The patient was then continued on oral prednisone acetate at 60 mg daily and mycophenolate mofetil for immunomodulatory treatment. After 3 months, the titers of anti-IgLON5 antibody in both the cerebrospinal fluid and blood significantly decreased. Polysomnography indicated a marked improvement in insomnia and apnea conditions, and cognitive function largely returned. Most encouragingly, 18F-FDG PET-CT scans revealed that the previously observed increased FDG uptake in the medulla oblongata and reduced FDG uptake in the left temporal lobe had normalized post-treatment. However, there are still many shortcomings. For example, although clinical follow-up extended for 1-year, crucial longitudinal laboratory data–including serial cerebrospinal fluid and serum analyses–were not available beyond the initial 3-months period due to patient-related reasons, precluding assessment of IgLON5 antibody titer dynamics. Moreover, the PET/CT findings are not broadly generalizable given the modality’s high cost and limited accessibility. Additionally, the observed hippocampal atrophy on MRI suggests a possible underlying neurodegenerative comorbidity, which may have confounded both the immune profile and the treatment response.

## 4 Conclusion

Anti-IgLON5 disease is a complex and rare form of autoimmune encephalitis characterized by sleep disturbances, cognitive dysfunction, psychiatric symptoms, movement disorders, autonomic dysfunction, and bulbar symptoms. The disease can progress to severe neurological impairment and, in severe cases, may lead to death. Our article presents a clinically classic case of anti-IgLON5 encephalitis, further substantiating the clinical manifestations of the disease, with a particular focus on central sleep apnea-hypopnea syndrome. Through immunomodulatory therapy, we observed significant improvement in the patient’s clinical symptoms. Additionally, we found that immunomodulatory treatment can markedly reduce the titers of anti-IgLON5 antibodies in both cerebrospinal fluid and blood. The use of 18F-FDG PET-CT to assess the efficacy of immunotherapy and the severity of the disease offers a novel perspective in the diagnosis and treatment of anti-IgLON5 disease. Overall, anti-IgLON5 antibody-associated disease is a multi-systemic condition requiring multidisciplinary management, necessitating further multicenter, large-sample studies to refine early diagnostic and identification methods and to seek precise and effective therapeutic strategies. This will significantly advance our comprehension of the disease’s natural progression and facilitate the establishment of standardized, multidisciplinary therapeutic protocols.

## Data Availability

The original contributions presented in this study are included in this article/Supplementary material, further inquiries can be directed to the corresponding authors.
