# Supplementary material for: Case Report: PET/CT assessment of immunomodulatory therapy in anti-IgLON5 encephalitis with sleep apnea
Source: Front Neurosci. 2026 Feb 23;20:1720844. doi: 10.3389/fnins.2026.1720844 (PMC12968195; doi:10.3389/fnins.2026.1720844)
Supplement: Supplementary file 1 [file Data_Sheet_1.docx]

Supplementary Material

# Supplementary Figure


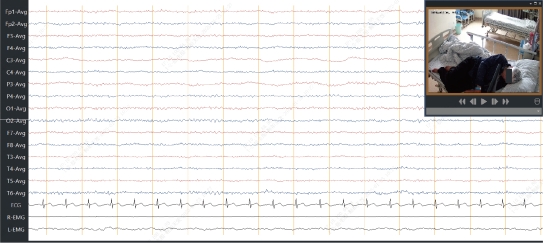


**Supplementary Figure 1.** Electroencephalogram. There were no significant abnormalities in the EEG, and no typical interictal epileptiform discharges.
